# Variants in Neuropeptide Y Receptor 1 and 5 Are Associated with Nutrient-Specific Food Intake and Are Under Recent Selection in Europeans

**DOI:** 10.1371/journal.pone.0007070

**Published:** 2009-09-17

**Authors:** Clara C. Elbers, Carolien G. F. de Kovel, Yvonne T. van der Schouw, Juliaan R. Meijboom, Florianne Bauer, Diederick E. Grobbee, Gosia Trynka, Jana V. van Vliet-Ostaptchouk, Cisca Wijmenga, N. Charlotte Onland-Moret

**Affiliations:** 1 Complex Genetics Section, Department of Biomedical Genetics, University Medical Center Utrecht, Utrecht, the Netherlands; 2 Julius Center for Health Sciences and Primary Care, University Medical Center Utrecht, Utrecht, the Netherlands; 3 Department of Pathology and Laboratory Medicine, University Medical Center Groningen, University of Groningen, Groningen, the Netherlands; 4 Department of Genetics, University Medical Center Groningen, University of Groningen, Groningen, the Netherlands; VU University Medical Center and VU University, the Netherlands

## Abstract

There is a large variation in caloric intake and macronutrient preference between individuals and between ethnic groups, and these food intake patterns show a strong heritability. The transition to new food sources during the agriculture revolution around 11,000 years ago probably created selective pressure and shaped the genome of modern humans. One major player in energy homeostasis is the appetite-stimulating hormone neuropeptide Y, in which the stimulatory capacity may be mediated by the neuropeptide Y receptors 1, 2 and 5 (*NPY1R, NPY2R* and *NPY5R*). We assess association between variants in the *NPY1R, NPY2R* and *NPY5R* genes and nutrient intake in a cross-sectional, single-center study of 400 men aged 40 to 80 years, and we examine whether genomic regions containing these genes show signatures of recent selection in 270 HapMap individuals (90 Africans, 90 Asians, and 90 Caucasians) and in 846 Dutch bloodbank controls. Our results show that derived alleles in *NPY1R* and *NPY5R* are associated with lower carbohydrate intake, mainly because of a lower consumption of mono- and disaccharides. We also show that carriers of these derived alleles, on average, consume meals with a lower glycemic index and glycemic load and have higher alcohol consumption. One of these variants shows the hallmark of recent selection in Europe. Our data suggest that lower carbohydrate intake, consuming meals with a low glycemic index and glycemic load, and/or higher alcohol consumption, gave a survival advantage in Europeans since the agricultural revolution. This advantage could lie in overall health benefits, because lower carbohydrate intake, consuming meals with a low GI and GL, and/or higher alcohol consumption, are known to be associated with a lower risk of chronic diseases.

## Introduction

One major player in energy homeostasis is the appetite-stimulating hormone neuropeptide Y (NPY) [1]. In rodents, NPY evokes eating behavior, inducing particularly carbohydrate intake. Injection of NPY in the brain elicits a strong feeding response even in satiated animals, eventually leading to obesity [2].

The effect of NPY is mediated by the neuropeptide Y receptors (NPYRs) [3]. Especially the Y1, Y2, and Y5 receptors (NPY1R, NPY2R, NPY5R) appear to be candidates for mediating the appetite stimulatory capacity of NPY[4], [5] through binding of NPY. These are receptors in the arcuate and paraventricular nuclei of the hypothalamus. Variants in genes coding for these receptors may therefore influence energy intake, which could influence an individual's susceptibility to becoming obese and developing T2D. We have previously pinpointed *NPY1R, NPY2R* and *NPY5R* as positional candidate genes for both obesity and T2D [6].

Large variations in caloric intake and macronutrient preference between individuals have been reported and these food intake patterns show a strong heritability [7]. There are also large differences in food intake and percentage of nutrient-specific energy intake among different ethnic groups [8], [9]. These ethnic differences in total and nutrient-specific energy intake might be caused by the natural selection of mutations providing an advantage for a particular environment or type of agriculture. The transition to different food sources during the agricultural revolution, which started around 11,000 years ago, was an important selective pressure and the changes in food intake helped shape the genome of modern humans [10]. Genome-wide sequence and SNP data of living humans can be used to study the recent natural selection over the past 30,000 years [11], [12]. Under neutral selection, the linkage disequilibrium (LD) around variants in the genome will decay over time due to recombination, so that older (common) alleles typically have short-range LD and younger (rare) alleles have long-range LD. However, when an allele is under positive selection, its frequency rises rapidly in the population over a short time span and the haplotype carrying the advantageous allele therefore breaks down more slowly than an allele with the same frequency under neutral selection.

In this study we investigated the role of single nucleotide polymorphisms (SNPs) in *NPY1R, NPY2R* and *NPY5R* genes in the total and nutrient-specific energy intake in a Dutch study population of 400 healthy older men. To see whether changing environments in the past may have caused adaptation or maladaption to our current life style, we examined whether these loci showed a signature of recent selection, using genome-wide SNP data from the HapMap populations and a genome-wide SNP dataset of 846 Dutch bloodbank controls.

## Results

### *NPY1R*, *NPY2R* and *NPY5R* Variation and Macronutrient Intake in Healthy Older Men

**Figure 1:**
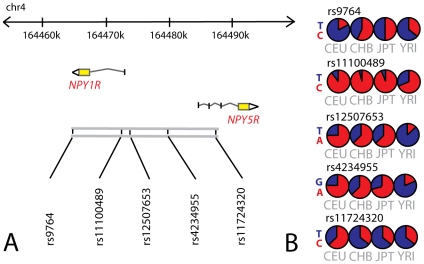
Characteristics of the *NPY1R/NPY5R* region. Figure 1A. tSNPs that optimally cover the genetic variation in the haplotype containing the *NPY1R* and *NPY5R* genes so that all SNPs with a minor allele frequency of ≥0.10 were captured with r^2^≥0.8. Figure 1B. The global allele frequency distributions per SNP are shown. *NPY1R* neuropeptide Y receptor 1; *NPY5R* neuropeptide Y receptor 5; CEU Utah residents with Northern and Western European ancestry from the CEPH collection; CHB Han Chinese in Beijing, China; JPT Japanese in Tokyo, Japan; YRI Yoruba in Ibadan, Nigeria.

Five tSNPs in the NPY2R gene and another five in the *NPY1R* and *NPY5R* genes (figure 1) were genotyped in the Hamlet population. The genotype success rates for all ten tSNPs were above 95%. There were no discordances in the genotypes of any of the CEPH sample and all genotypes were in agreement with Hardy-Weinberg equilibrium (p>0.01). Age, body mass index (BMI) and macronutrient intake of the participants are shown in Table 1. As neither age nor BMI were associated with any of the *NPY2R* and *NPY1R/NPY5R* SNPs, they do not confound the relation in this study. Therefore they were not included as covariates in the model.

**Table 1:** Characteristics of the Hamlet study population.

Characteristic	N	Mean (SD)
Age (years)	382	60.40 (11.22)
BMI (kg/m2)	382	26.27 (3.44)
Energy intake (kcal)	380	2255.30 (517.48)
Protein intake (% of total energy intake)	380	15.02 (2.01)
Fat intake (% of total energy intake)	380	35.59 (5.09)
Carbohydrate intake (% of total energy intake)	380	42.98 (6.64)
Alcohol intake (% of total energy intake)	380	6.41 (6.46)

BMI body mass index; SD standard deviation.

We did not find an association between any of the SNPs and total energy intake. However, by studying macronutrient-specific energy intake, we observed associations between SNPs in the *NPY1R/NPY5R* genes and carbohydrate intake, and with alcohol intake.

**Figure 2:**
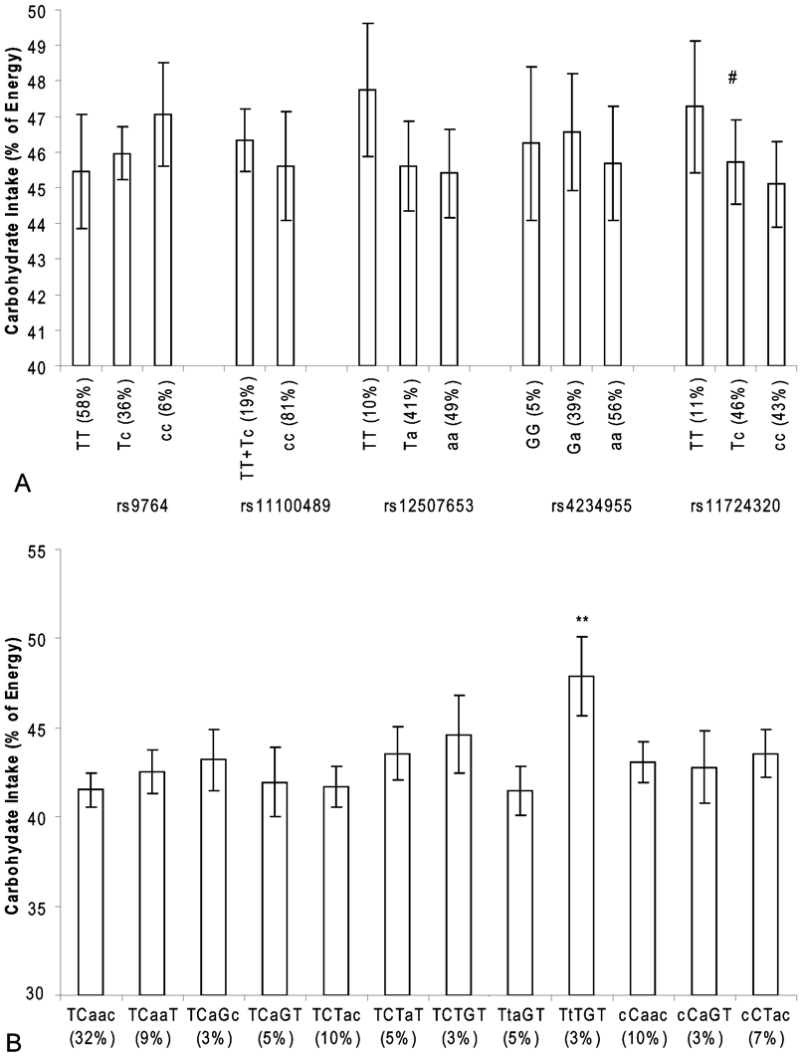
*NPY1R/NPY5R* variants and carbohydrate intake in the Hamlet population. Figure 2a shows the association of SNPs in the *NPY1R/NPY5R* region with carbohydrate intake as percentage of total energy intake. The ancestral alleles are indicated as capital letters. # p<0.01 for trend. Figure 2b shows the association of *NPY1R/NPY5R* haplotypes with carbohydrate intake as percentage of total energy intake. The haploblocks consist of the SNPs rs9764, rs11100489, rs12507653, rs4234955 and rs11724320 and the ancestral alleles are indicated as capital letters. **p<0.01 (compared with linear regression model).

For rs17724320 in the *NPY1R/NPY5R* genes, we found a dose-response relationship of the derived T allele with carbohydrate intake (p<0.01 for trend), meaning that carbohydrate intake was lowest in men carrying two ancestral C alleles and that it increased with each extra derived allele (Figure 2a). The haplotype analysis showed that carriers of the TTTGT haplotype consumed, on average, 6.2% more total carbohydrates than carriers of the reference haplotype TCAAC (p = 0.003) (Figure 2b).

**Figure 3:**
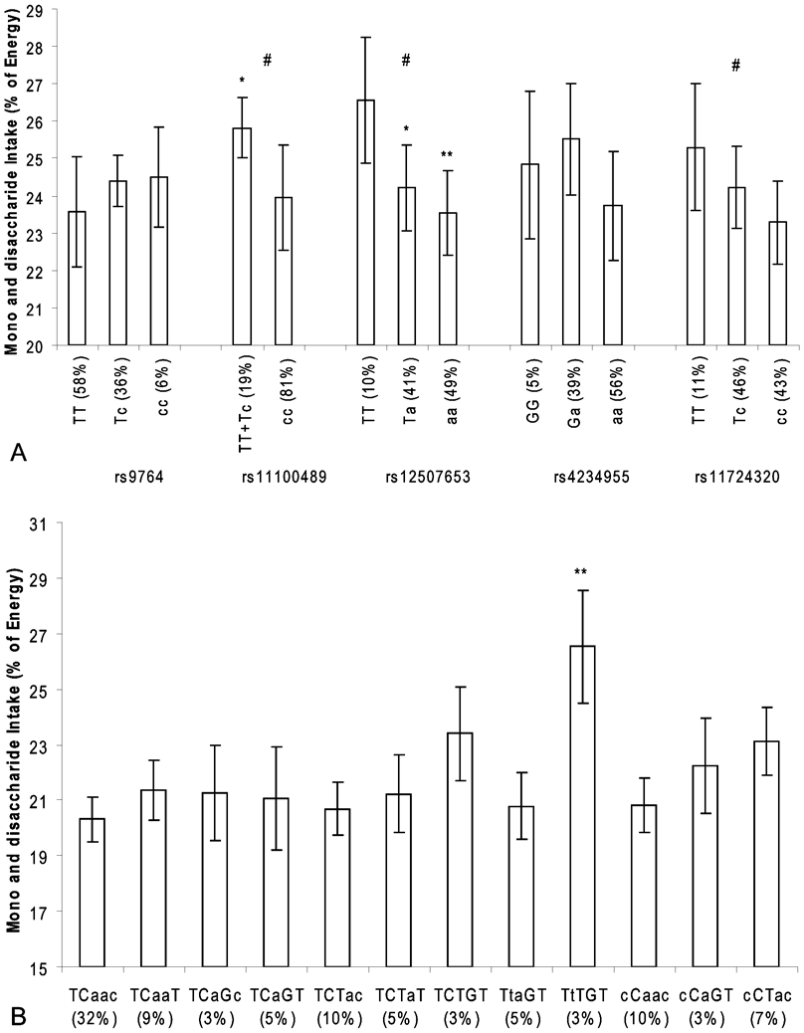
*NPY1R/NPY5R* variants and mono- and disaccharide intake in the Hamlet population. Figure 3a shows the association of SNPs in the *NPY1R/NPY5R* region with mono- and disaccharide intake as percentage of total energy intake. The ancestral alleles are indicated as capital letters.*p<0.05, **p<0.01(compared with linear regression model), # P<0.01 for trend. Figure 3b shows the association of *NPY1R/NPY5R* haplotypes with mono- and disaccharide intake as percentage of total energy intake. The haploblocks consist of the SNPs rs9764, rs11100489, rs12507653, rs4234955 and rs11724320 and the ancestral alleles are indicated as capital letters. *p<0.05 (compared with linear regression model).

**Figure 4:**
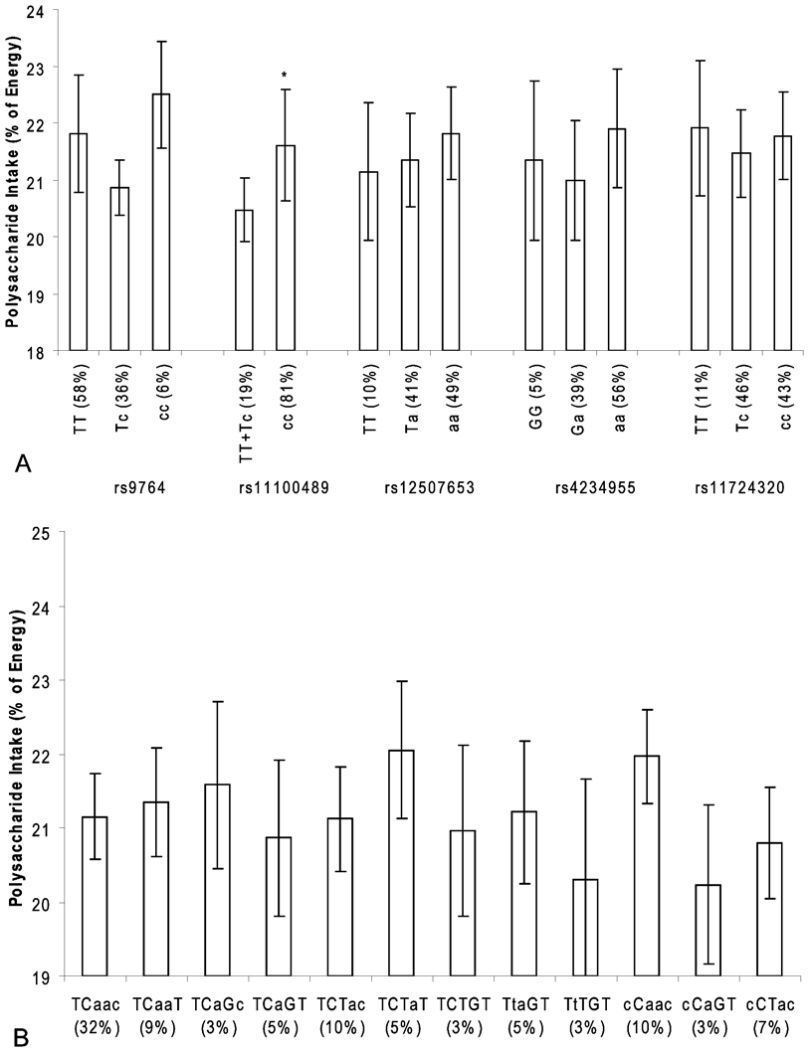
*NPY1R/NPY5R* variants and polysaccharide intake in the Hamlet population. Figure 4a shows the association of SNPs in the *NPY1R/NPY5R* region with polysaccharide intake as percentage of total energy intake. The ancestral alleles are indicated as capital letters.*p<0.05(compared with linear regression model) Figure 4b shows the association of *NPY1R/NPY5R* haplotypes with polysaccharide intake as percentage of total energy intake. The haploblocks consist of the SNPs rs9764, rs11100489, rs12507653, rs4234955 and rs11724320 and the ancestral alleles are indicated as capital letters. *p<0.05 (compared with linear regression model).

There are many types of carbohydrates and the physiological responses to these vary substantially. We therefore also studied the association between SNPs in the *NPY1R/NPY5R* genes and relative mono- and disaccharide intake, relative polysaccharide intake, and GI and GL. The association appeared to be mainly restricted to mono- and disaccharides. For rs11100489, rs12507653, rs4234955 and rs17724320 in the *NPY1R/NPY5R* genes, we found the same dose-response relationship with mono- and disaccharide intake as for total carbohydrates for the derived allele (all showed a p<0.05 trend) (Figure 3a). Men carrying two derived C alleles of rs11100489 ate 1.9% less mono- and disaccharides compared to men carrying one or two ancestral T alleles (p = 0.02). For rs12507653, men carrying one or two derived A alleles consumed 2.3% and 3.0% less mono- and disaccharides, respectively, than men homozygote for the ancestral T allele (p = 0.04 and p = 0.008, respectively). For rs11100489, the same genotype that was associated with a decrease in mono- and disaccharide intake was also associated with an increase in polysaccharide intake of 1.1% (p = 0.05) (Figure 4a).

The haplotype analysis showed that carriers of the TTTGT haplotype consumed 6.2% more mono- and disaccharides than carriers of the reference TCAAC haplotype (p = 0.002) (Figure 3b). We found no difference in polysaccharide intake between the different haplotypes (Figure 4b).

**Figure 5:**
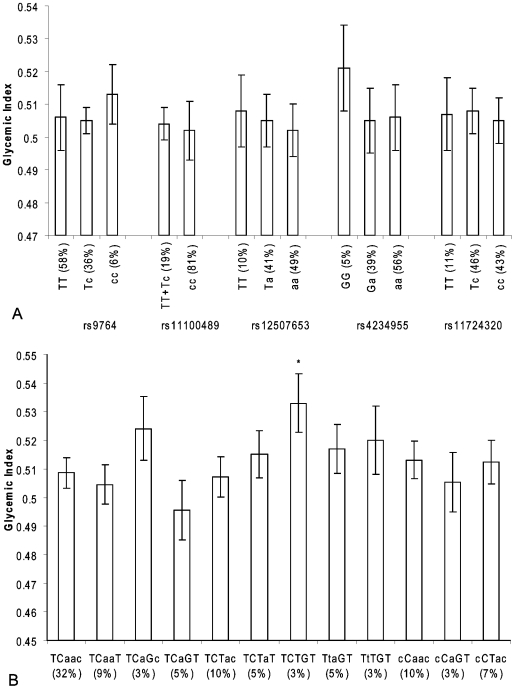
*NPY1R/NPY5R* variants and dietary glycemic index in the Hamlet population. Figure 5a shows the association of SNPs in the *NPY1R/NPY5R* region with dietary glycemic index. The ancestral alleles are indicated as capital letters. Figure 5b shows the association of *NPY1R/NPY5R* haplotypes with dietary glycemic index. The haploblocks consist of the SNPs rs9764, rs11100489, rs12507653, rs4234955 and rs11724320 and the ancestral alleles are indicated as capital letters. *p<0.05 (compared with linear regression model).

**Figure 6:**
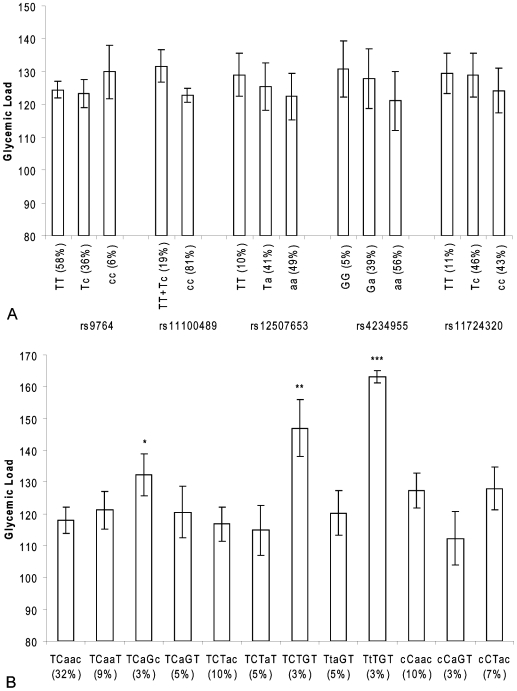
*NPY1R/NPY5R* variants and dietary glycemic load in the Hamlet population. Figure 6a shows the association of SNPs in the *NPY1R/NPY5R* region with dietary glycemic load. The ancestral alleles are indicated as capital letters. Figure 6b shows the association of *NPY1R/NPY5R* haplotypes with dietary glycemic index. The haploblocks consist of the SNPs rs9764, rs11100489, rs12507653, rs4234955 and rs11724320 and the ancestral alleles are indicated as capital letters. *p<0.05, **p<0.001, ***p<0.0001 (compared with linear regression model).

There were no associations between single SNP genotypes and GI and GL (Figures 5a and 6a). However, the GI of the daily food intake of individuals carrying the TCTGT haplotype was significantly higher than of individuals carrying the reference haplotype TCAAC (0.533 versus 0.509; p = 0.01) (Figure 5b). The daily food intake of individuals who carry the ancestral TCTGT, TCAGC or TTTGT haplotypes had a significantly higher GL than individuals carrying the reference haplotype TCAAC (147, 160 and 130 versus 118, respectively; p = 0.001, p = 0.03 and p<0.0001, respectively)) (Figure 6b).

**Figure 7:**
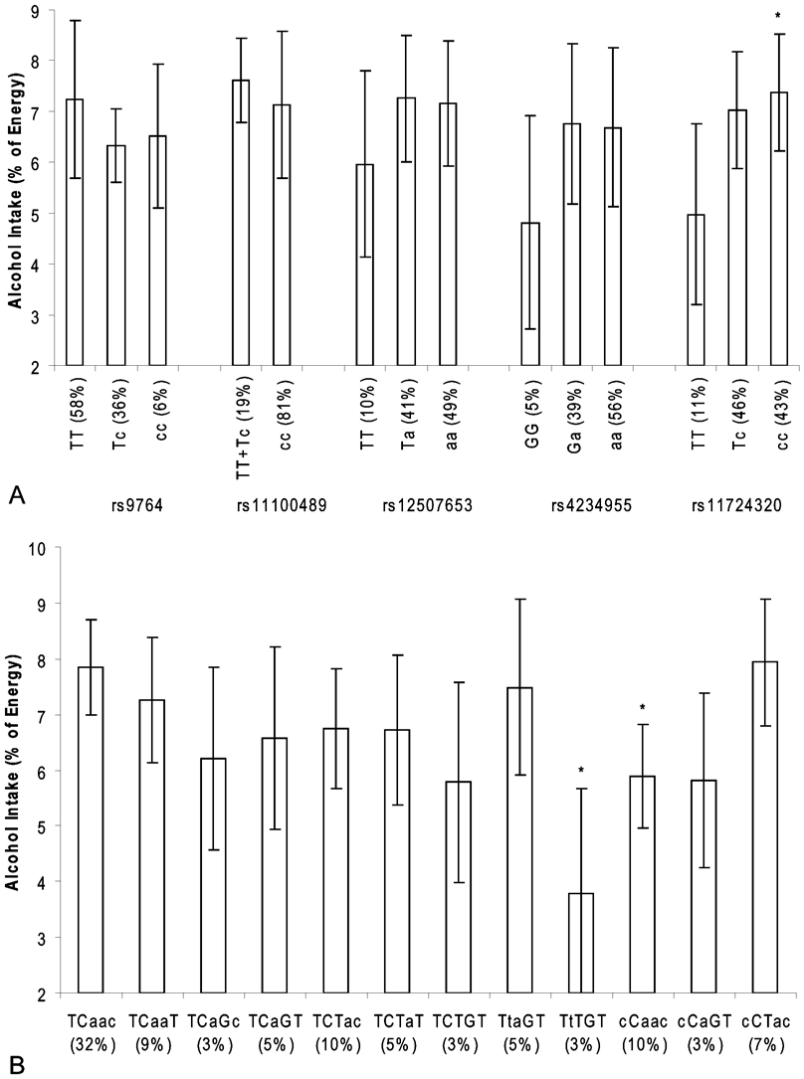
*NPY1R/NPY5R* variants and alcohol intake in the Hamlet population. Figure 3a shows the association of SNPs in the *NPY1R/NPY5R* region with alcohol intake as percentage of total energy intake. The ancestral alleles are indicated as capital letters. *p<0.05, **p<0.01(compared with linear regression model), # P<0.01 for trend. Figure 3b shows the association of *NPY1R/NPY5R* haplotypes with alcohol intake as percentage of total energy intake. The haploblocks consist of the SNPs rs9764, rs11100489, rs12507653, rs4234955 and rs11724320 and the ancestral alleles are indicated as capital letters. *p<0.05 (compared with linear regression model).

For alcohol intake there was an association with rs11724320 in the *NPY1R/NPY5R* genes (Figure 7a). Men homozygote for the derived allele consumed 2.4% more alcohol than men homozygote for the ancestral allele (p = 0.04). Carriers of the TTTGT and CCAAC haplotypes showed a difference of 4.0% and 2.0% in relative consumption of alcohol compared to the reference haplotype TCAAC (both p-values: 0.03) (Figure 7b).

### Signatures of Recent Selection in the *NPY1R/NPY5R* Gene

**Figure 8:**
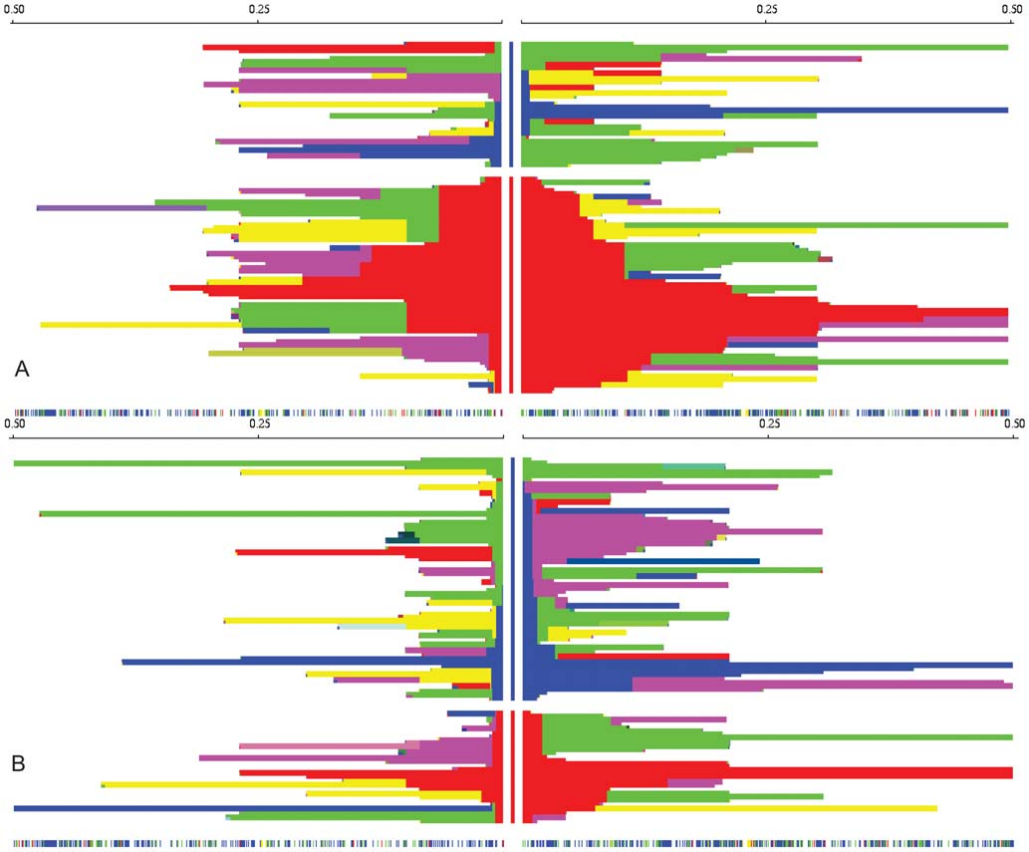
Haplotype decay around rs11724320 in Europeans and Africans. Figure 8a shows the haplotype decay in genomic region of 1Mb around rs11724320, located in the *NPY1R/NPY5R* region, in 90 Utah residents with Northern and Western European ancestry from the CEPH collection (European HapMap individuals). Each horizontal line represents a haplotype and the center column represents the core SNP rs11724320 with the derived C-allele in red and the ancestral T-allele in blue. The derived C allele of rs11724320is positioned on an unusually long haplotype compared to the ancestral T allele in the European HapMap individuals. Figure 8b shows the haplotype decay in genomic region of 1Mb around rs11724320, located in the *NPY1R/NPY5R* region, in 90 YRI Yoruba in Ibadan from Nigeria (African HapMap individuals). The haplotype lengths around the derived C allele and the ancestral T allele of rs11724320 do not significantly differ in the African population.

As evident from Figure 8a, the derived C allele of rs11724320, located in the *NPY1R/NPY5R* region, is positioned on an unusually long haplotype compared to the ancestral T allele in the European HapMap individuals. In the African and Asian HapMap individuals, the haplotype lengths around rs11724320 are much shorter and there is no difference in haplotype lengths between the derived locus and the ancestral locus (Figure 8b: Africans).

The standardized iHS score is −2.160 inHapMap Caucasians, indicating that the haplotypes on the derived allele background are significantly longer than the haplotypes associated with the ancestral allele (empirical p-value p = 0.03). This indicates that the locus is under recent positive selection in the European HapMap population.

**Figure 9:**
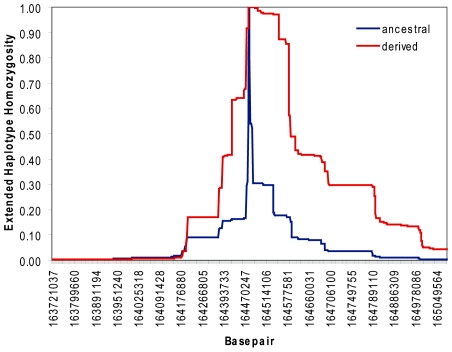
Haplotype decay around rs11724320 in 864 Caucasians from the Netherlands. Extended haplotype homozygosity and distance from core region rs11724320 in GWAS data with the derived allele shown in red and the ancestral allele in blue. For this analysis we used the derived and ancestral haplotypes of 864 Caucasians from the Netherlands. Although the derived C allele is very common in this population (allele frequency of 63%), it has long-range LD. This implies that the allele frequency increased rapidly in the population over a short time span.

To replicate these results, we calculated the haplotype decay of the derived and the ancestral alleles around the same SNP in 846 Caucasians, using EHH and iHS analysis. In this population, the derived C allele frequency was 63% and that for the ancestral T allele 37%. Although the derived C allele is very common in Europeans, it has long-range linkage disequilibrium (Figure 9). The standardized iHS score is −2.12 in for the *NPY1R/NPY5R* locus in the Dutch dataset and this correlates with a empirical p-value of 0.05. This implies that the allele frequency rose rapidly in the population over a short period and it confirms our previous findings that the C allele of rs11724320 is under positive selection in Europeans.

### Age Estimation of Locus under Selection

We used the haplotype of the 846 Dutch Caucasians to obtain a crude estimate of the time of the selective sweep. At EHH = 0.25, the haplotype length around the derived allele of rs11724320 is 436,713 base pairs long. The number of generations *g* therefore equals (ln 0.25/−2*0.436713)*100≈160. Taking the generation time to be 25 years, the ancestor time becomes *t* = 25 *g* and the selective sweep therefore started 25*160≈4000 years ago. The support interval, calculated at EHH = 0.15 and EHH = 0.35 is ∼3800 to ∼4300 years ago.

## Discussion

The results of this study show that derived alleles in *NPY1R* and *NPY5R* are associated with lower relative carbohydrate intake, mainly because of a lower consumption of mono- and disaccharides. We also show that carriers of these derived alleles on average consume meals with a lower GI and GL. However, the same alleles are associated with increased alcohol consumption. The derived allele of rs11724320 appears to be under recent selection in the European population, and probably originates from around 4,000 years ago.

A predicted selective sweep of around 4,000 years ago fits the theory of adaptation to novel food sources during the agriculture revolution, which started in Europe around 6,000 years ago and was gradually further developed from that point on. Our data suggest that a lower carbohydrate intake, consumption of meals with a low GI and GL, and/or higher alcohol consumption gave a survival advantage in Europeans during the agricultural revolution.

### Adaptation to New Food Sources

The consumption of new food sources or the transition to novel dietary habits can lead to selective pressure when certain gene variants are better adapted to a particular dietary habit than others, resulting in a survival advantage for humans carrying the better adapted variants of the gene. One famous example is the selective advantage of variants in the lactase gene (*LCT*) which preserves the ability to digest lactose, the major sugar in milk, after weaning and throughout adult life [13]. This lactase persistence is considered an adaptation to dietary change brought about by the development of agriculture and animal domestication and husbandry.

The agricultural revolution started around 11,000 years ago in the area of the Black Sea and was accompanied by major changes in diet for many human populations [14]. In Western Europe, agriculture was started about 6,000 years ago and was gradually further developed from that point on. Therefore the selection of the derived allele of rs11724320, which originated about 4,000–5,000 years ago, might well have been driven by the transition to novel food habits.

The Mediterranean agriculture that developed in Europe at that time comprised livestock husbandry, which supplied much more protein and fat than the agricultures that developed in warmer parts of the world [14]. It can be argued that the allele under selection in the *NPY1R/NPY5R* genes, which is associated with reduced carbohydrate intake, is in fact adapted to this increased amount of protein and fat in the diet after the agriculture revolution. A higher percentage of dietary protein and fat necessarily results in a lower percentage of other macronutrients like carbohydrates.

However, we did not find an association between SNP variants in the *NPY1R/NPY5R* genes and fat and protein intake as a percentage of total energy intake, even when we took the percentage of fat and protein together in the linear regression model. This could be due to power-related issues, because one limitation of our study is the small size of the Hamlet population. Since, however, a reduced relative carbohydrate uptake must be compensated for by increased fat, protein and alcohol intake, we cannot exclude the possibility that the selective pressure was in fact due to an increased use of fat and proteins rather than reduced (simple) sugar consumption.

Thrifty genes, favoring the economical use and storage of energy, confer a survival advantage in times of food scarcity [15]. Currently, these thrifty genes are maladapted to our ‘Westernized’ diet and lifestyle and may nowadays be contributing to the occurrence of obesity and T2D worldwide. However, it is possible that Europeans had already started adapting genetically to a ‘Westernized’ diet with high fat and protein intake after the rise of Mediterranean agriculture. The lower frequency of T2D in Europeans compared to other ethnic groups that are now adopting a ‘Westernized’ diet and lifestyle supports this hypothesis [16].

An alternative hypothesis is that new food sources may have helped Europeans to adapt to colder climates with less sunlight [17]. Ultraviolet radiation (UVR) can damage the bare human skin, but it is also important for the synthesis of vitamin D. This vitamin plays an essential role in the mineralization and normal growth of bone during infancy and childhood. Apart from the lighter skin pigmentation, a demand for adequate vitamin D synthesis in the less sunny northern European climate may therefore have favored adaptation to vitamin D deficiency with more consumption of high fat and high protein products like liver, fish, oils, eggs and milk products (these products contain vitamin D) [17]. Caucasians living in Western Europe may have required efficient thermogenesis to cope with cold climates. Lipids from fat, but not glucose, contribute to thermogenesis during exposure to cold [18]. However, a recent study that investigated the Y chromosome and mitochondrial DNA (both parts of the DNA which do not show recombination) in 2000 Dutch men showed that 80% originated from hunter-gatherers that already populated Western Europe 25,000 year ago (http://www.nrcnext.nl/nieuws/wetenschap/article2030713.ece, article in Dutch). The ancestors of modern residents of the Netherlands had already lived in Europe long before the agriculture revolution began and had probably already adapted to colder climates with less sunlight.

Another possibility is that instead of a survival advantage for high fat and high protein intake, the selective pressure may have been due to a survival advantage for low-carbohydrate and/or low GI/GL diets. Many studies have assessed the effectiveness and safety of different weight-loss diets, including the low-carbohydrate diet without calorie restriction [19]–[21]. After 12 to 48 months, the participants who were on the low-carbohydrate diet not only showed significantly more weight-loss compared to participants in other diet groups, but also experienced positive changes in overall metabolic effects and lipid profiles. They also experienced a decrease in C-reactive protein levels and in blood pressure. Thus, reducing carbohydrate intake as a percentage of total energy intake results in overall health benefits, even though the total energy intake is not restricted.

All dietary carbohydrates can be digested or converted into glucose. However, there are several types of carbohydrates (like monosaccharides, disaccharides, oligosaccharides, and starch and non-starch polysaccharides) and people's physiological glycemic and insulinemic responses to these different carbohydrates vary substantially [22]. A high GI meal is followed by rapid absorption of glucose and rapid stimulation of insulin secretion and other hormones. Within the first 2 hours of consuming a high GI meal, plasma glucose levels can become twice as high as after consuming a low GI meal containing identical nutrition and energy. This rapid response after ingestion of a high GI meal challenges the mechanism of energy homeostasis; acute metabolic effects follow a high GI meal [22].

A meta-analysis of observational studies on the effects of dietary GI and GL on the risk of chronic diseases showed an association of high-GI and/or high-GL with an increased risk of chronic diseases, such as type 2 diabetes, coronary heart disease, gall bladder disease, and breast cancer [23].

Finally, as we also found an increased alcohol intake for the derived allele carriers, we cannot exclude the possibility that increased alcohol consumption had a survival advantage. Rodent studies indicate that ethanol consumption and resistance are inversely related to NPY signaling. Both the NPY- and NPY1R-deficient mice showed increased ethanol consumption and reduced sensitivity to ethanol-induced sedation [24]. Multiple studies in humans show that moderate alcohol consumption has a protective effect for T2D possibly due to increased insulin sensitivity [25], [26]. Also, anti-inflammatory effects of moderate alcohol consumption may be involved in this risk reduction [27].

### NPY1R/NPY5R - The Hypothalamus Pathway and Nutrient-Specific Food Intake

To our knowledge this is the first report of variants in the NPY1R/NPY5R genes being associated with nutrient-specific food intake. Our findings correspond with rodent studies in which NPY evoked feeding behavior, inducing particularly carbohydrate intake.

Two other genes from the hypothalamus pathway also have been found to be associated with nutrient-specific food intake, but not with total energy intake. The Ala67Thr SNP in the agouti-related protein (AGRP) gene was associated with lower fat intake and higher carbohydrate intake[28] and the rs2272382 SNP in the *TUB* gene was shown to be associated with an increased energy intake from carbohydrates, mainly because of consuming more mono- and disaccharides. The same SNP was shown to be associated with a higher daily GL food intake [29]. This implies that the hypothalamus pathway plays an important role in controlling nutrient-specific food intake.

### Correcting for multiple testing

In this study the p-values of the results of the association analysis are presented without correction for multiple testing. We justify this firstly, because we do not test hypothesis-free. Secondly, both the macronutrient intake (in percentage of total energy intake) and the tagging SNPs are not independent measurements.

In the first stage of the analyses we tested 5 SNPs in *NPY2R* and 5 SNPs in *NPY1R/NPY5R* region for association with macro-nutrient intake (as percentage of total energy intake). In the second stage of the analyses we continued with the most interesting findings and therefore we tested 5 SNPs and 12 haplotypes in the *NPY1R/NPY5R* region for association with subgroups of carbohydrate intake (mono- and disaccharides, polysaccharides, GI and GL).

In the first stage, after controlling for testing 10 SNPs for association with macronutrient intake by the False Discovery Rate (FRD) procedure none of the associations remain statistically significant at a threshold (q) of 0.10. However in the second stage, after controlling for testing 5 SNPs and 12 haplotypes in the *NPY1R/NPY5R* region for association with subgroups of carbohydrate intake, the association of haplotype TTTGT with carbohydrate intake, the associations of haplotype TTTGT and rs12507653 with mono- and disaccharides and the associations of haplotypes TTTGT, TCTGT and TCAGA and GL remain statistically significant at a threshold (q) of 0.10.

Further studies should be done to confirm these associations in other populations.

### Conclusion

We show that derived alleles in *NPY1R* and *NPY5R* are associated with lower carbohydrate intake, mainly because of a lower consumption of mono- and disaccharides. We also show that carriers of these derived alleles, on average, consume meals with a lower glycemic index and glycemic load and have higher alcohol consumption. One of these variants shows the hallmark of recent selection in Europe.

Our data suggest that lower carbohydrate intake, consuming meals with a low glycemic index and glycemic load, and/or higher alcohol consumption, gave a survival advantage in Europeans since the agricultural revolution. This advantage could lie in overall health benefits, because lower carbohydrate intake, consuming meals with a low GI and GL, and/or higher alcohol consumption, are known to be associated with a lower risk of chronic diseases.

## Methods

### Study Populations

#### Hamlet study

The Hamlet study is a cross-sectional, single-center study in 400 men aged 40 to 80 years living independently. The recruitment of the participants has been described elsewhere [30]. In brief, participants visited the study center twice for physical examinations, including drawing of blood, and filled in a validated food frequency questionnaire (FFQ) on their dietary intake, which is designed to estimate regular intake of 178 food items in the year before enrolment [31], [32]. We calculated and assigned the values (grams/day) for total energy, fat, carbohydrates, protein and alcohol for each food item in the FFQ (described in detail by de Kleijn *et al.* [33]). Energy-adjusted intake was calculated using the nutrient-density method [34].

We calculated glycemic load (GL) by multiplying the glycemic index (GI) of a food item with its carbohydrate content, then multiplied this value with its frequency of consumption and summed the values over all food items [35], [36]. Glycemic load thus represented both quality and quantity of carbohydrates, and interaction between the two. Each unit of dietary glycemic load represented the equivalent of 1 g carbohydrate from glucose. The overall glycemic index of a man's diet was calculated by dividing the dietary glycemic load by the total amount of carbohydrate consumed. Such expression of dietary glycemic index per gram of carbohydrate thus reflects the overall quality of the daily carbohydrate intake.

The Pearson correlation coefficient between the FFQ and twelve monthly recall questionnaires (each for a 24-hour period) ranged from 0.61 to 0.85 for the macronutrients, energy intake and alcohol intake.

All participants gave written informed consent before enrolment and the study was approved by the institutional review board of the University Medical Center Utrecht. Data collection took place between March 2001 and April 2002.

#### HapMap

A total of 270 people are included in the HapMap database (Phase II) [36]: 30 trios of US residents with Northern and Western European ancestry (CEU), 30 trios of Yoruba people from Ibadan, Nigeria (YRI), 45 unrelated Japanese individuals from the Tokyo area (ASN), and 45 unrelated Chinese individuals from Beijing (ASN).

#### GWAS data

We used a genome-wide dataset of 846 Dutch blood bank controls. More details on this study are described elsewhere [37]. All individuals gave their informed consent. This study was approved by the Medical Ethical Committee of the University Medical Center Utrecht.

### Genotyping in Hamlet

Information about SNPs in the *NPY1R, NPY2R, NPY5R* genes was obtained from the HapMap project (www.hapmap.org, HapMap data Rel#21/phase II Jul 06). Tagging SNPs (tSNPs) were selected using Haploview version 3.2, which is based on Tagger software (www.broad.mit.edu/mpg/tagger/) [38], so that all SNPs with a minor allele frequency (MAF) of ≥0.10 were captured with r^2^≥0.8. We selected five tSNPs (rs6849115, rs1021868, rs12507396, rs1047214, rs9990860) for the NPY2R gene and five more (rs9764, rs11100489, rs12507653, rs4234955 and rs17724320) for the *NPY1R* and *NPY5R* genes, as these two genes are located together in the human genome (Figure 1).

These SNPs were genotyped in the Hamlet study using Taqman assays-on-demand (Applied Biosystems, Nieuwerkerk a/d IJssel, the Netherlands), performed according to the manufacturer's specifications. The sequence information for all primers and probes is available upon request. The genotypes were analyzed using a TaqMan 7900HT (Applied Biosystems, Nieuwerkerk a/d IJssel, the Netherlands). The DNA samples were processed in 384-well plates. Each plate contained 8 negative controls and 16 genotyping controls, which consisted of four duplicates of four different samples obtained from the Centre d'Etude du Polymorphisme Humain (CEPH).

### Data Analysis in Hamlet

The genotype frequencies were tested for Hardy–Weinberg equilibrium by χ^2^ analysis. Association between genotypes (as the independent variable) and macronutrient intake (as dependent variables) was determined using linear regression analysis. We studied single SNP associations with total energy intake and macronutrient-specific energy intake using the ancestral allele as reference in the linear regression model, although it was not always the most frequent allele. The ancestral allele was based on alignment to the chimpanzee sequence. As we wished to study the effect on macronutrient intake independent of total energy intake, this total intake was included in the models as an explanatory variable. We also performed trend analyses to test a dose-response effect for the derived alleles.

The False Discovery Rate (FDR) method from Benjamini and Hochberg was used to control for multiple testing [39].

All statistical analyses were performed using SPSS, version 15.0 for Windows (SPSS, Chicago, IL, USA). Haplotype analyses in Hamlet were performed using the haplo.stats package of R (version 2.7.1). The ancestral haplotype had an allele frequency of 0.03 in the Hamlet population and we therefore included all allele frequencies >0.02 for analysis. The TCAAC haplotype was the most common, with an allele frequency of 0.33, and this haplotype was used as a reference in the linear regression model. We did not use the ancestral haplotype as a reference because of its low frequency in the Hamlet population.

### Integrated Haplotype Score (iHS) Analysis in

We used the web-based tool haplotter to calculate extended haploblocks around our SNPs in HapMap and the online available software to calculate extended haploblocks around SNPs in our genome wide dataset, using the iHS method [12]. iHS is a statistic that was developed to detect evidence of recent positive selection (<30,000 years ago) at a locus, and is based on the differential levels of linkage disequilibrium surrounding a positively selected allele compared to the background allele at the same position. An extremely positive iHS score (>2) means that haplotypes on the ancestral allele background are longer than the derived allele background, while an extremely negative iHS score (<−2) means that the haplotypes on the derived allele background are longer than the haplotypes associated with the ancestral allele.

### Extended Haplotype Homozygosity Analysis in the GWAS Data

A region of 1 Mb around *NPY1R/NPY5R* was extracted from the imputed GWAS dataset. We used the Beagle software program to infer haplotypes from genotypes of the Dutch subjects [40]. Then we calculated haplotype decay around the SNPs in the *NPY1R/NPY5R* region by performing extended haplotype homozygosity (EHH) analysis, using R (version 2.7.1). An EHH value stands for the probability that all haplotypes are homozygote at a recombination distance *r* from the selected site. We started by choosing the ancestral allele of the core SNP; at this point EHH = 1 for that allele. Next we compared the ancestral allele of the core SNP with the first proximate SNP upstream and looked for the most frequent haplotype between the ancestral allele of the core SNP and each of the alleles of the first proximate SNP. This meant that all individuals with the most frequent haplotype remained for analysis; all individuals with the other haplotype were excluded forever. If, for example, 80% of the individuals showed the most frequent haplotype between the ancestral allele and the first proximate SNP upstream, then EHH = 0.80 at that point. Subsequently, the first proximate allele upstream was compared with the second one and the same comparison and inclusion was done. We repeated this analysis with all proximate alleles on the upstream side of the ancestral allele of the core SNP until all subjects were excluded (EHH = 0) and we performed the same procedure with all alleles located downstream from the core SNP. We then repeated this whole procedure for the derived allele of the core SNP.

### Age Estimation

We used the data from the EHH analysis of the 864 Dutch individuals to obtain a crude estimate of the age of expansion of the derived variant as a result of recent selection, the so-called selective sweep. For this analysis we assumed a star phylogeny of the haplotypes. The recombination distance *r* is the distance in cM/Mbp between EHH = *x* to the left of the core SNP and EHH = *x* to the right of the core SNP. For a chosen *x, r* can be obtained from the data. As both *x* and *r* are then known, the generation time *g* can be calculated as: g = (ln x/−2r)* 100. Assuming an average generation length of 25 years, the age of the selective sweep equals 25 *g*. For this study, we calculated *r* at EHH  = 0.25 (support interval EHH = 0.15−EHH = 0.35).
